# Inhibition of RhoA GTPase and the subsequent activation of PTP1B protects cultured hippocampal neurons against amyloid β toxicity

**DOI:** 10.1186/1750-1326-6-14

**Published:** 2011-02-04

**Authors:** Pedro J Chacon, Rosa Garcia-Mejias, Alfredo Rodriguez-Tebar

**Affiliations:** 1 Centro Andaluz de Biología Molecular y Medicina Regenerativa (CABIMER), Americo Vespucio s/n, Isla de la Cartuja, 41092 Seville, Spain

## Abstract

**Background:**

Amyloid beta (Aβ) is the main agent responsible for the advent and progression of Alzheimer's disease. This peptide can at least partially antagonize nerve growth factor (NGF) signalling in neurons, which may be responsible for some of the effects produced by Aβ. Accordingly, better understanding the NGF signalling pathway may provide clues as to how to protect neurons from the toxic effects of Aβ.

**Results:**

We show here that Aβ activates the RhoA GTPase by binding to p75^NTR^, thereby preventing the NGF-induced activation of protein tyrosine phosphatase 1B (PTP1B) that is required for neuron survival. We also show that the inactivation of RhoA GTPase and the activation of PTP1B protect cultured hippocampal neurons against the noxious effects of Aβ. Indeed, either pharmacological inhibition of RhoA with C3 ADP ribosyl transferase or the transfection of cultured neurons with a dominant negative form of RhoA protects cultured hippocampal neurons from the effects of Aβ. In addition, over-expression of PTP1B also prevents the deleterious effects of Aβ on cultured hippocampal neurons.

**Conclusion:**

Our findings indicate that potentiating the activity of NGF at the level of RhoA inactivation and PTP1B activation may represent a new means to combat the noxious effects of Aβ in Alzheimer's disease.

## Background

According to the amyloid hypothesis, amyloid beta (Aβ) aggregates form deposits in the brain, the process that precipitates the different manifestations of Alzheimer's disease (AD) [1]. Consequently, most therapeutic approaches to treat AD centre on this peptide: on the one hand attempting to limit the production of Aβ or the formation of fibrils and aggregates [2,3], while on the other hand, favouring its clearance. Therapeutic approaches aimed at clearing Aβ plaques have received special attention, and methods for active or passive immunisation have proven effective in reducing Aβ content in the brain. Nevertheless, these strategies have failed to conclusively ameliorate or retard cognitive deterioration in AD patients [4,5].

Another approach that could be considered involves blocking the signals induced by Aβ that provoke neuronal death. However, despite extensive studies into the effects of Aβ on neurons, our understanding of Aβ signalling remains fragmented, and a consistent framework for such processes has yet to be defined. Still, recent publications have reinforced the notion that Aβ interferes with insulin signalling [6] and indeed, when soluble forms of Aβ bind to dendrites, they provoke the removal of insulin receptors (probably by activating their internalization), as well as preventing synapse formation [7]. In addition, intracellular Aβ may impair insulin signalling by preventing phosphoinositide-dependent kinase dependent activation of Akt [8]. This Aβ-promoted disruption of insulin signalling has prompted clinical trials in which insulin activity is primed and stimulated [9,10].

By contrast, Aβ neurotoxicity has also been associated with the trophic effects of NGF. Indeed, some therapeutic approaches for AD involve the use of NGF or mimic the effects of NGF [11-16]. Indeed, the cellular and molecular bases underlying the antagonism of NGF by Aβ were recently elucidated in part. Aβ competes with NGF for binding to p75^NTR ^[17,18], thereby preventing the activation of NF-κ-B by impairing the tyrosine phosphorylation and subsequent degradation of I-κ-B [19]. The inhibition of NF-κ-B promoted by Aβ results in the downregulation of *Homologous of Enhancer-of-split 1 *(*Hes1*) expression, a gene that has an important influence on dendrite patterning and GABAergic inputs [20,21].

In this study, we show that Aβ impairs the initial steps of NGF signalling at the level of the RhoA GTPase and PTP1B. We also show that potentiating NGF signalling by inhibiting RhoA GTPase and activating PTP1B offers cells certain resistance against Aβ neurotoxicity.

## Results

### Aβ (1-42) induces morphological changes and regulates neuron survival via p75^NTR^/RhoA

**Figure 1:**
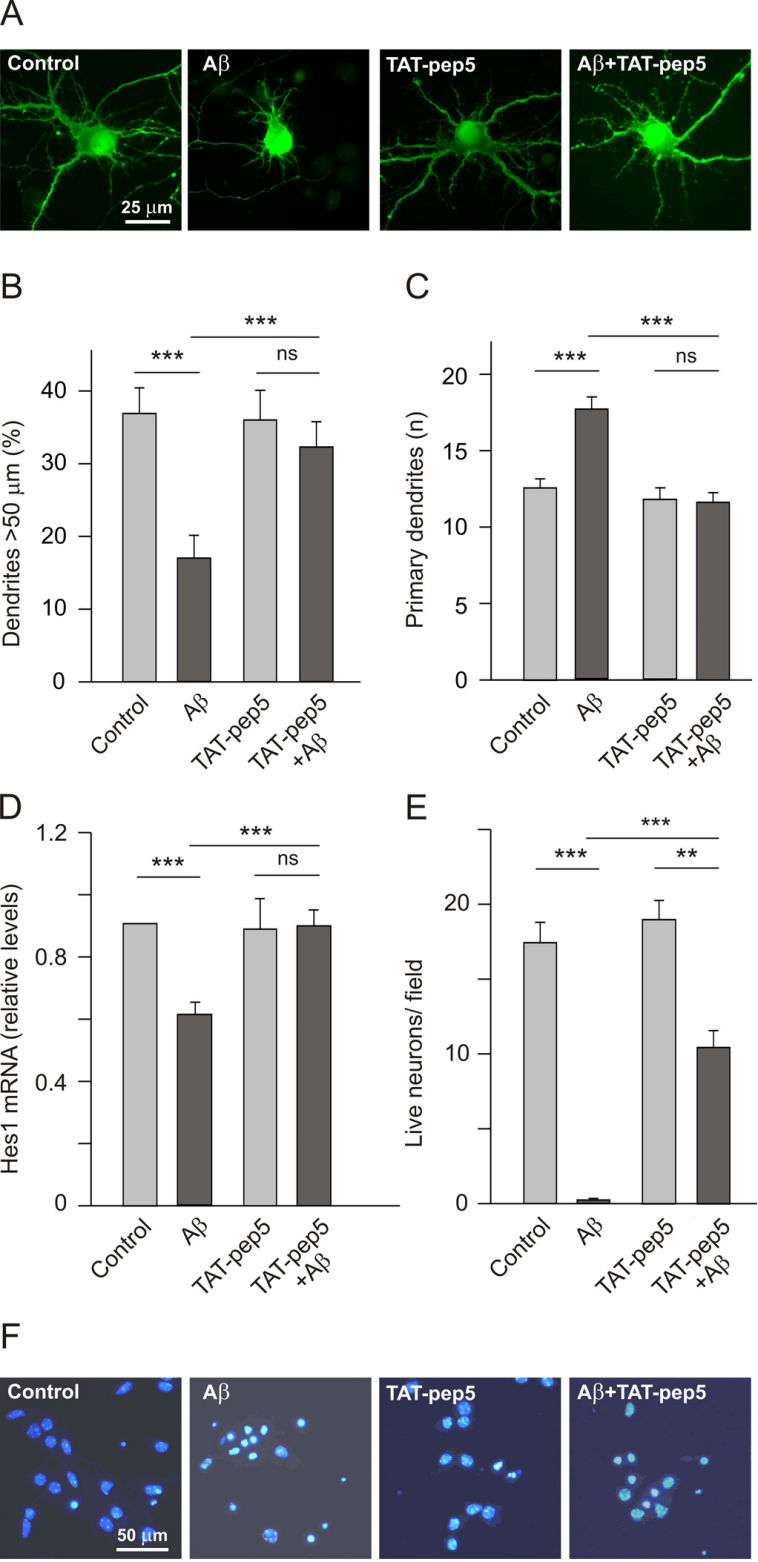
**TAT-pep5 specifically uncouples p75^NTR ^from the RhoA GTPase, and it counteracts the effects of Aβ on dendrite patterning, gene expression and the survival of cultured hippocampal neurons**. (A, B and C) E17 hippocampal neurons were plated at a density of 40,000 cells/cm^2 ^and cultured for 7 DIV. Neurons were transfected with pEGFP and then exposed for a further 16 h to TAT-Pep5 (1.0 μM), Aβ (5 μM), or both. The cells were fixed and labelled with the anti-EGFP antibody, and then processed for immunofluorescence. (A) representative micrographs of cultured neurons under the different conditions. The relative dendrite length (B) and primary dendrite numbers (C) was quantified as indicated in the methods section. Note that TAT-pep5 reversed the effects of Aβ on dendrite length and number. (D) Neurons cultured for 7 DIV (40,000 cells/cm^2^) were first incubated with TAT-Pep5 (1.0 μM) for 18 h, after which they were stimulated with Aβ (5 μM) for 4 h, lysed and then processed for real time PCR to quantify *Hes1 *expression. Note that TAT-pep5 prevented the Aβ-induced decrease in Hes1 mRNA. (E) 7 DIV cultures (30,000 cells/cm^2^) were stimulated with TAT-pep5 (1.0 μM) and/or Aβ (5 μM) for 90 h. The neurons were then stained with DAPI and those with intact nuclei were counted. Note that TAT-pep5 rescued around half of the neurons from the deleterious effects of Aβ. (F) Representative micrographs of DAPI stained nuclei in cultured hippocampal neurons treated with Aβ, or with Aβ and TAT-pep5, the latter conferring resistance against Aβ.

Previous studies revealed that Aβ binds to p75^NTR ^receptors [17,18], and more recent data indicates that p75^NTR ^mediates the toxic effects of amyloid on cholinergic neurons [22,23]. Our earlier studies [19] showed that Aβ may specifically influence the morphology, gene expression and survival of cultured hippocampal neurons. Indeed, exposure of these neurons to Aβ (5 μM for 16 h) increased the number of primary dendrites they emitted, while restricting their length (Figure 1A, B and 1C). However, when the intracellular activity of p75^NTR ^was specifically uncoupled by incubating these neurons with TAT-pep5 (1.0 μM) [24,25], the influence of Aβ on the neurons' morphology was abolished (Figure 1A, B and 1C). Accordingly, Aβ no longer caused an increase in dendrite number nor did it diminish their length in the presence of TAT-pep5. We also showed previously that the expression of Hes1 mRNA decreases when hippocampal neurons are exposed to Aβ (5 μM for 4 h: Figure 1D). Since Hes1 mRNA transcripts augment in the presence of NGF (100 ng/ml) [20], the loss of these transcripts suggests that amyloid reverses the effects of NGF [19]. In accordance with the morphological effects observed in these neurons, pharmacological inhibition of p75^NTR ^signalling with TAT-pep5 prevented Aβ from inhibiting *Hes1 *expression. This is particularly significant because *Hes1 *over-expression protects cells against the deleterious effects of Aβ, and preventing the decrease of *Hes1 *expression improves the survival of neurons exposed to Aβ (Chacon et al., unpublished results). Perhaps most importantly, application of TAT-pep5 (1.0 μM) protected hippocampal neurons from the death induced by prolonged exposure to Aβ (5 μM for 90 h: Figure 1E and 1F). Thus, these data indicate that by interacting with the p75^NTR^/RhoA signalling pathway, Aβ combats the positive effects of NGF in terms of morphology, gene expression and survival.

### The role of RhoA in the neurotoxicity of Aβ

**Figure 2:**
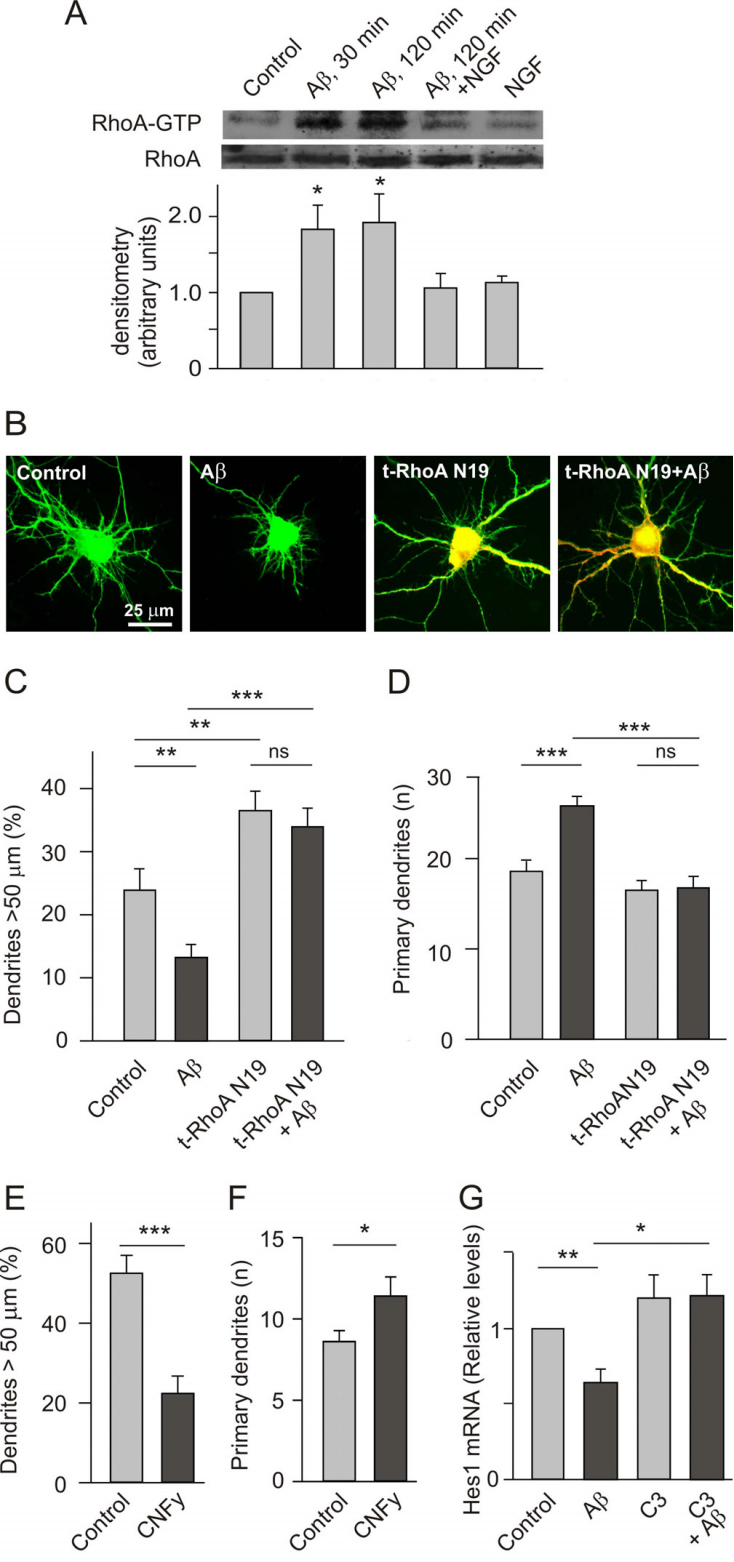
**Aβ activates RhoA thereby influencing neuron morphology**. (A) Western blots showing the activated and total RhoA GTPase in extracts from cultured PC12 nnr5 cells stimulated with NGF (100 ng/ml) for 5 h at the times indicated, or with Aβ (5 μM). Note that in contrast to NGF, Aβ increased the levels of RhoA GTP. The quantification of RhoA GTP in the lower panel is an average from four independent experiments. (B) Representative micrographs of hippocampal neurons cultured for 7 DIV (40,000 cells/cm^2^), treated with Aβ (5 μM) and/or co-transfected with EGFP and a myc tagged RhoA N19 (a *dn *form of RhoA) for 16 h. (C, D) Quantification of relative dendrite length (C) and primary dendrite number (D) in the four conditions indicated. Note that the attenuation of RhoA GTPase activity counteracted the effects of Aβ on dendrite length and number. Also note in (C) that the attenuation of RhoA activity increased the length of dendrites *per se*. (E, F) Quantification of relative dendrite length (E) and primary dendrite number (F) in cultured neurons after addition of CNFy (200 ng/ml: a specific activator of RhoA) for 16 h. (G) 7 DIV neurons in culture were first incubated with C3 ADP rybosyl transferase (1.0 μM) for 18 h, they were stimulated with Aβ (5 μM) for 4 h, lysed and then processed for real time PCR to quantify *Hes1 *expression. Note that the inhibition of RhoA by C3 prevented the Aβ-induced decrease in Hes1 mRNA.

Since TAT-pep5 prevents p75^NTR ^from associating with Rho-GDI intracellularly, thereby blocking its ability to activate RhoA GTPase [24,25], we assessed to what extent RhoA might mediate the effects of Aβ. In cultured PC12 nnr5 cells (cells devoid of TrkA), activated RhoA was largely increased within 2 h of exposure to Aβ (5 μM) (Figure 2A). By contrast, incubation with NGF (100 ng/ml for 5 h) hardly decreased the levels of activated RhoA, probably because such levels were already low in this experimental system. However, the addition of NGF prevented the activation of RhoA induced by Aβ (5 μM for 2 h). These results suggested that the activation of RhoA induced by Aβ through p75^NTR ^may be important for Aβ to influence the morphology and survival of cells. To test this hypothesis, we studied the role of RhoA in mediating the effects of Aβ on dendrite patterning. Thus, the transfection of hippocampal neurons with a dominant negative form of RhoA, RhoA N19 [26], counteracted the effects of Aβ on dendrite patterning (Figure 2B, C and 2D). When specific parameters of dendrites were quantified (Figure 2C and 2D), Aβ failed to decrease dendrite length or increase the number of primary dendrites when the activity of RhoA was abrogated by transfection of the *dn *form of this GTPase. By contrast, activation of RhoA for 16 h by applying 200 ng/ml CNFy to cultured neurons [27] produced changes in dendrite patterning, such as a decrease in dendrite length and an increase in dendrite number (Figure 2E and 2F). Further evidence that the inhibition of RhoA to some extent interrupted Aβ signalling in neurons was obtained when neurons were treated with a cell permeable form of the C3 ADP ribosyl transferase (1.0 μM, 18 h). Inhibition of RhoA prevented Aβ from impeding *Hes1 *expression (Figure 2G) in a similar manner to the effects of TAT-pep5 on neurons (Figure 1D).

**Figure 3:**
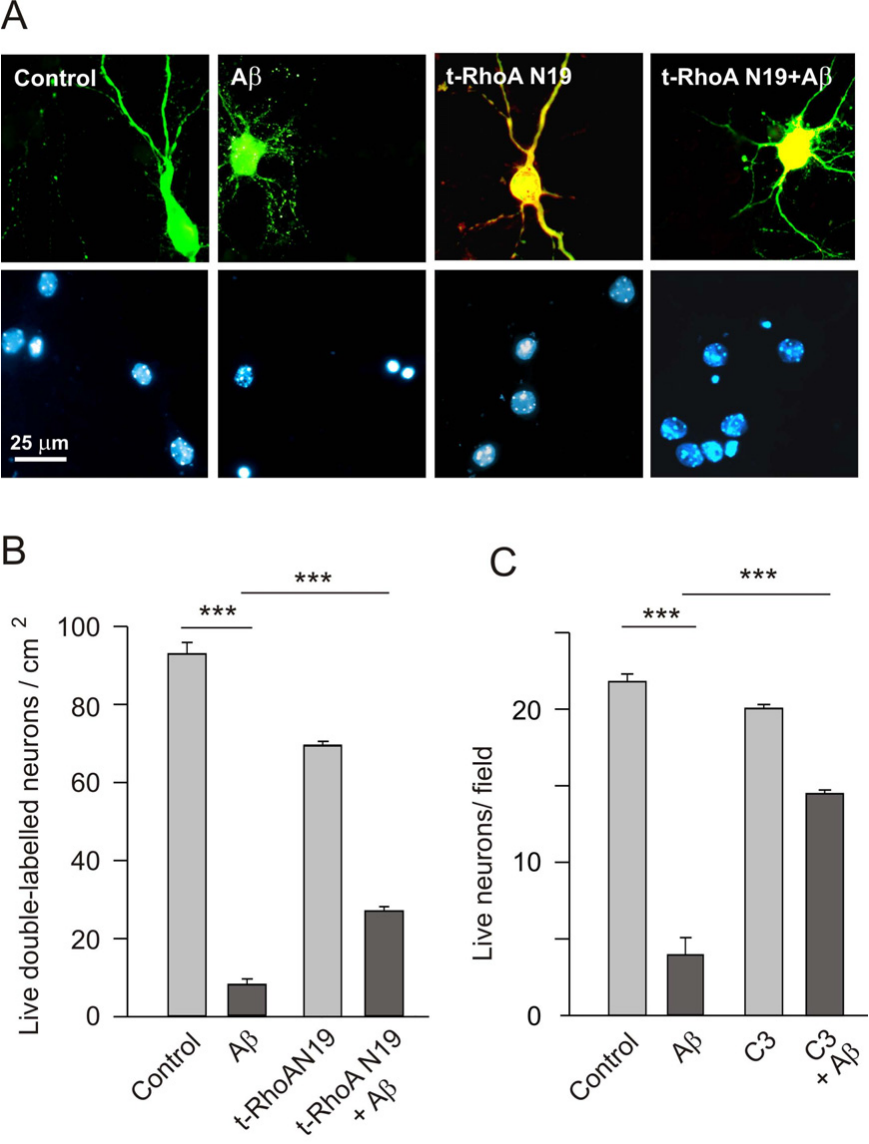
**The role of RhoA in Aβ induced neuron death**. (A, B) Hippocampal neurons (30,000 cells/cm^2^) were cultured for 7 days and then treated with Aβ (5 μM). Two days later the neurons were transfected with the *dn *RhoA N19 and on the following day, the cells were stained and the number of live cells were determined as described in the Methods. (A) Representative micrographs of double-labelled cultured hippocampal neurons under the four conditions described. Green represents EGFP immunostaining, red is the transfected myc-tagged RhoA N19 and the DAPI stained nuclei are blue. (B) Quantification of live cells. Note that transfection with the dominant negative form of RhoA rescued a significant number of neurons from Aβ-induced death. (C) The effects of Anti-amyloid were more dramatic when C3 ADP ribosyl transferase (1 μM), a RhoA inhibitor, was applied to the cultures. Cultured hippocampal neurons (7 DIV) were treated simultaneously with C3 ADP ribosyl transferase and Aβ, and the number of live cells was determined four days later in culture.

RhoA GTPase not only exerts an important influence on neuron morphology but also, on survival of neurons treated with Aβ. Indeed, inhibition of RhoA partially protected neurons against the noxious effects of Aβ and transfection of the *dn *form of RhoA also increased the cells' resistance to Aβ neurotoxicity (Figure 3A and 3B). Likewise, prior exposure of the cultured neurons to C3 ADP ribosyl transferase (1.0 μM) made neurons resistant to Aβ (5 μM: Figure 3C), further evidence of the role of RhoA in Aβ induced neuron death. However, treating neurons with CNFy for 90 h did not produce cell death (data not shown), indicating that the noxious effects of Aβ are not exclusively mediated by RhoA.

### Protein tyrosine phosphatase 1B activity protects neurons against Aβ neurotoxicity

**Figure 4:**
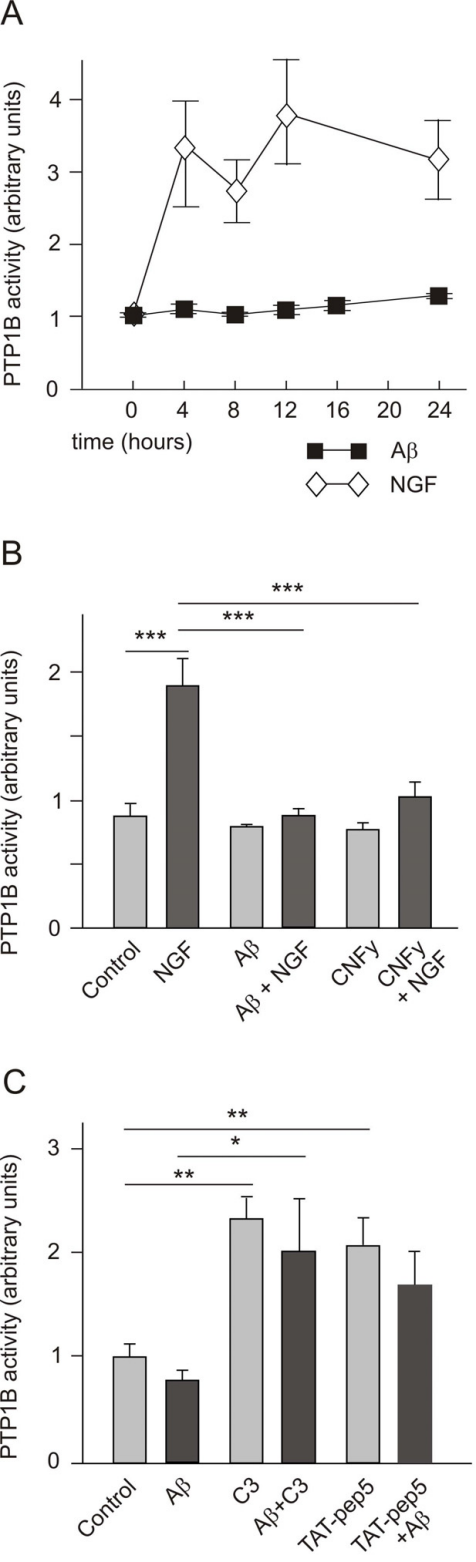
**Amyloid β interferes with the capacity of NGF to activate protein tyrosine phosphatase 1B in hippocampal neurons**. Cultured 7 DIV neurons (about 250,000 cells per experimental point) were treated as indicated and PTP1B activity was assessed. (A) Whereas NGF (100 ng/ml) increased PTP1B activity several fold, Aβ (5 μM) did not alter the activity of the phosphatase, although Aβ did prevent the increase of PTP1B activity induced by NGF (B). Another activator of RhoA activity, CNFy (200 ng/ml), also prevented the activation of PTP1B caused by NGF. Both Aβ and CNFy were applied to cultures 18 h before stimulating with NGF for 4 h. (C) The inhibition of RhoA activity after incubating the cells with either C3 (1 μM) or TAT-pep5 (1 μM) for 18 h increased the activity of PTP1B. By contrast to NGF, such increases were not counteracted by prior application of Aβ.

It is thought that PTP1B activity was controlled by the RhoA GTPase, although the precise mechanism involved remains unknown [28]. Since NGF increases PTP1B activity by binding to p75^NTR ^[29], we tested whether PTP1B might participate in Aβ signalling in hippocampal neurons. Indeed, while NGF (100 ng/ml) increased the activity of PTP1B four-fold, Aβ (5 μM) failed to do so (Figure 4A). Moreover, prior application of Aβ to cultured hippocampal neurons prevented NGF from activating this phosphatase (Figure 4B). When PTP1B activity was assessed in extracts from cells grown under different conditions, it was at least partially controlled by RhoA. Thus, the treatment of neurons with the selective activator of the RhoA GTPase, CNFy (200 ng/ml) [27], also prevented NGF from activating the phosphatase (Figure 4B). Since the activation of RhoA by either Aβ or CNFy prevented PTP1B activation, we concluded that RhoA is a negative regulator of the phosphatase. In fact, inhibiting RhoA with C3 ADP rybosyl transferase was sufficient to increase PTP1B activity, which interestingly could not be prevented by amyloid. Furthermore, application of TAT-pep5 to the cultured neurons also increased PTP1B activity and again, this was not prevented by Aβ (Figure 4C). Together, these results demonstrate that PTP1B activity was mediated by p75^NTR^/RhoA and more specifically, that p75^NTR^, a receptor for both NGF and Aβ, plays an important role in the regulation of this phosphatase.

**Figure 5:**
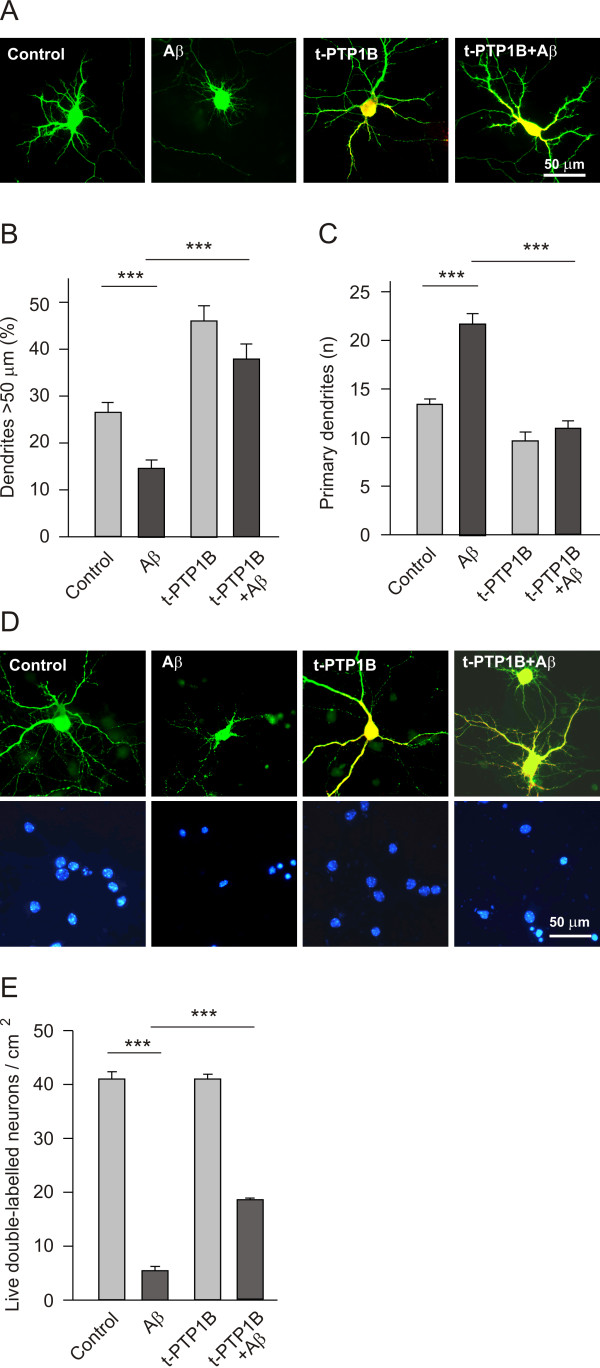
**Overexpression of PTP1B counteracts the effects of Aβ on dendrite patterning (A, B, C) and neuron death (D, E)**. Cultured hippocampal neurons (40,000 cells/cm^2^, 7 DIV) were co-transfected with the EGFP and PTP1B plasmids, treated with Aβ (5 μM) and incubated for a further 16 h to analyse dendrite patterning (A, B, C). (A) Representative micrographs of cultured hippocampal neurons at 7 DIV treated with Aβ and/or transfected with PTP1B. EGFP immunostaining is in green and the transfected HA-tagged PTP1B in red. (B, C) Quantification of the relative dendrite length (B) and primary dendrite number (C) in the four conditions indicated. Note that overexpression of PTP1B increased dendrite length and prevented the morphological effects of Aβ. (D, E) Hippocampal neurons (30,000 cells/cm^2^) were cultured for 7 days and then treated with Aβ (5 μM). Two days later, the neurons were transfected with the PTP1B expressing plasmid, and on the following day the cells were stained and the live cells determined as described in the Methods. (D) Representative micrographs of double-labelled cultured hippocampal neurons under the four conditions described. EGFP immunostaining is in green, the transfected HA-tagged PTP1B in red and the DAPI stained nuclei are blue. (E) Quantification of live cells. Note that transfection with the PTP1B expressing plasmid rescued a significant number of neurons from Aβ-induced neuron death.

The effect of Aβ on PTP1B was further evident when the morphology and survival of neurons over-expressing PTP1B was studied. In terms of morphology, overexpression of PTP1B mimics the effects of NGF on dendrite patterning [20] and most importantly, it counteracts the effects of Aβ. The overexpression of PTP1B increased the length of dendrites and counteracted the shortening of dendrites caused by Aβ (Figure 5A and 5B). In addition, the increase in dendrite number when neurons were exposed to Aβ was prevented in neurons overexpressing PTP1B (Figure 5A and 5C). However, the effects of PTP1B activity on survival were even more dramatic, as overexpression of PTP1B rescued about half of the neurons from the death caused by Aβ (Figure 5D and 5E). Taken together these results demonstrate that neurons require an active form of PTP1B to survive. Furthermore, these data also indicate that impairment of NGF by Aβ induces PTP1B activation, which plays an important role in the induction of neuron death by Aβ.

## Discussion

### The effects of Aβ on cultured neurons mediated by p75^NTR^

The noxious effects of Aβ appear to be at least partially due to the neutralisation of NGF activity in neurons [19] (see also [30]) and indeed, we demonstrated that Aβ prevents the activation of NF-κ-B and the increase of *Hes1 *expression caused by NGF. We also more recently revealed novel features of NGF signalling in neurons, including the activation of PTP1B after binding to p75^NTR ^[29], that is in turn necessary for the tyrosine phosphorylation and degradation of I-κ-B, and the subsequent activation of NF-κ-B. p75^NTR ^appears to be the only receptor mediating the effects of NGF on both RhoA and PTP1B. Indeed, we showed that a selective blockage of the receptor prevented NGF-induced PTP1B activation and that the inactivation of TrkA did not abolish such an effect [29].

The participation of p75^NTR ^in the activity of Aβ is well documented, and radioactive Aβ was seen to bind to the receptor and trigger apoptosis in cell lines that overexpress p75^NTR ^[17,18]. More recently, neurons expressing a mutated form of p75^NTR ^devoid of its extracellular domain were shown to be resistant to the neurite dystrophy associated to c-Jun activation and to apoptosis, both in vitro and in vivo [23,31]. In addition, the fact that Aβ augments the expression of p75^NTR ^in SH-SY5Y human neuroblastoma cells is further evidence for the role of p75^NTR^, especially given that it also increases in hippocampal neurons from a transgenic mouse model of AD [32].

In a PC12 cell variant devoid of TrkA, we established that Aβ activates RhoA by binding to p75^NTR^, an effect that is partially prevented by exposing the cells to NGF prior to the administration of Aβ. Activation of RhoA by Aβ has also been observed in SH-SY5Y cells, in which it controls the phosphorylation of the collapsing response mediator protein-2, an effect that disrupts its binding to β-tubulin and neurite growth prior to cell death [33].

### The role of RhoA GTPase in neurodegeneration

There is increasing evidence that RhoA is an essential effector of certain neurodegenerative processes. The binding of various myelin associated ligands to the Nogo receptor complex, which includes p75^NTR^, produces RhoA activation [34]. Moreover, disruption of this receptor's function or deactivation of RhoA facilitates neurite and axonal growth in injured CNS neurons [35,36]. RhoA activity has also been associated to AD, particularly since the distribution of RhoA is altered in the brains of AD patients and in an AD mouse model. Moreover, activated RhoA augments in dystrophic dendrites and diminishes around synapses [37]. Here, we reveal that activation of RhoA not only decreases dendrite length but also, it is an important mediator of Aβ-induced neuron death. Indeed, activation of RhoA by CNFy mimics the effects of Aβ on dendrite morphology, although it did not mimic the deleterious effects of Aβ on the cells. Nevertheless, the role of RhoA in neuronal death was assessed since attenuating the GTPase activity, either by transfecting neurons with a *dn *form of RhoA or through pharmacological inhibition with C3 ADP ribosyl transferase, protects a significant number of neurons from Aβ neurotoxicity. These results indicate that RhoA activation plays a role in AD development, suggesting that inhibition of this GTPase might delay the progress of the disease.

### Relationship between RhoA and PTP1B

A functional analysis revealed that constitutively active RhoA inactivates PTP1B, although the precise mechanism underlying such inhibition remains unknown [28]. Our studies demonstrate a functional relationship between these two enzymes, since the pharmacological inhibition of RhoA was followed by activation of PTP1B, thereby mimicking the effects of NGF on the phosphatase. Conversely, the activation of RhoA by CNFy, a yersinia toxin [27] prevented NGF from activating PTP1B, an effect reminiscent of the action of Aβ. However, activation of RhoA did not diminish PTP1B activity below basal levels and the activation of RhoA only reduced the fraction of PTP1B activity increased by NGF. This may indicate that not all PTP1B molecules are functionally linked to the state of RhoA activation, reflecting the variety of signalling pathways in which PTP1B is involved [38].

RhoA controls dendrite length and hampers dendrite elongation, spine formation and synapse stabilization by a mechanism in which the activation of ROCK is involved [39]. The fact that RhoA also governs dendrite patterning by mechanisms that don't involve PTP1B may explain how CNFy decreases dendrite length without affecting the activity of the phosphatase. However, the true nature of the RhoA/PTP1B connection still remains unclear. Reasonable candidates to participate in this process are the reactive oxygen species (ROS) and indeed, early studies revealed that Aβ increased the ROS pool in neurons [40,41] reviewed in [42]. Increased levels of ROS may act in several ways, activating RhoA by oxidising a redox sensitive domain [43], or by inactivating cysteine phosphatases like PTP1B, as seen in vitro [44] and in cellular systems after calcium influx [45], as well as after application of insulin [46], EGF [47] or IL-4 [48].

### Activation of PTP1B is needed for neuron survival

PTP1B is often constitutively active although its activity may be negatively controlled by Akt induced serine phosphorylation of the phosphatase [49], tyrosine phosphorylation [50], or extracellular stimulation of ROS levels that oxidise the active centre of the phosphatase under the control of extracellular effectors such as insulin [51]. Activation of PTP1B activity is associated with the C-terminal cleavage of the enzyme [52], reviewed in [53]. However, activation of PTP1B associated to extracellular stimuli such as NGF has only recently been observed [29], and post-translational modifications of the enzyme that enhance its activity have yet to be identified.

**Figure 6:**
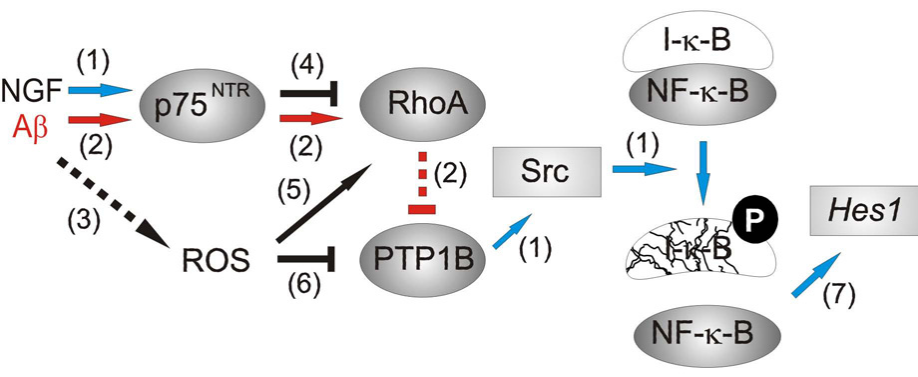
**Diagram showing the signals transduced by NGF leading to *Hes1 *expression and neuron survival, and their impairment by Aβ**. Various steps have been defined here and are shown in red (labelled as 2). The steps in black ((3), (4), (5) and (6)) come from the literature and the steps labelled in blue (1 and 7) are from our previous studies [19-21,29].

Excess PTP1B activity is associated with important pathologies such as type II diabetes, obesity [54,55] and tumorigenesis [53], which has driven the search for PTP1B inhibitors [56]. In particular, the impairment of insulin signalling by uncontrolled PTP1B activity has been correlated with the appearance of both type II diabetes and AD [57]. Thus, the use of PTP1B inhibitors that strengthen insulin signalling may prevent at least some of the problems associated with diabetes and hopefully, the development of AD. However, our results indicate that PTP1B lies in the NGF signalling pathway and that it is activated by this factor via RhoA inactivation. PTP1B activity is needed for neuron survival and it helps cultured hippocampal neurons resist the attack of amyloid. Our early studies show that PTP1B may activate the Src kinase, which is needed for NF-κ-B activation and *Hes1 *expression [29] (see Figure 6). Therefore, it would interesting to evaluate the neurological side-effects of the pharmacological inhibition of PTP1B in animal models.

## Conclusions

The mechanism by which we believe NGF promotes *Hes1 *expression and by which Aβ opposes such effects is outlined in Figure 6 in which the main findings of the present study are represented in red, our previous results are in blue and other published findings are in black. Thus, the main conclusions of the present studies are: (i) Aβ activates RhoA via p75^NTR^; (ii) the activation of RhoA inactivates PTP1B; (iii) inactivation of RhoA by either pharmacological inhibitor or transfection with a *dn *isoform makes neurons resistant to Aβ; (iv) increasing PTP1B activity makes neurons overexpressing the phosphatase resistant to Aβ. In summary, by strengthening different elements in the NGF signalling pathway it is feasible to make neurons more resistant to the effects of amyloid.

## Methods

### Antibodies

For immunocytochemistry, the primary antibodies used were a rabbit anti-enhanced green fluorescent protein (EGFP) from Invitrogen (Carlsbad, CA; used at a dilution of 1:500), a rat anti-hemagglutinin (HA) mAb and a mouse anti-Myc mAb (both used at a dilution of 1:400; Roche Applied Science, Mannheim, Germany). Western blots were probed with a mouse anti-RhoA (1:250; Santa Cruz Biotechnology, Santa Cruz, CA) and a mouse anti-PTP1B was used for immunoprecipitations (1:50; BD Transduction Laboratories, Lexington, KY). The goat anti-rabbit Cy^2 ^(1:1000), goat anti-rat Cy^3 ^(1:500), goat anti-mouse Cy^3 ^(1:1000) and donkey anti-mouse-HRP (1:5000) secondary antibodies were all obtained from Jackson Immuno Research (West Grove, USA).

### Other Chemicals

NGF from mouse salivary glands was obtained from Alomone Labs (Jerusalem, Israel), and it was used at a concentration of 100 ng/ml. Amyloid β (1-42) was obtained from NeoMPS (Strasbourg, France) and it was used at a concentration of 5 μM. This peptide was dissolved in 1,1,1,3,3,3-hexafluoro-2-propanol to obtain the oligomeric form of Amyloid β. After the solvent was allowed to evaporate for 2 hrs at room temperature, the peptide film was dissolved in DMSO, sonicated in a water bath for 10 min diluted to 100 μM in PBS and briefly vortexed, before it was incubated overnight at 4 °C. Aliquots were stored at -20°C. C3 ADP ribosyl transferase (Cytoskeleton Inc., Denver, CO), a cell permeable Rho inhibitor, was used at 500 ng/ml. CNFy (cytotoxic necrotizing factor from *Yersinia pseudotuberculosis*), a specific activator of RhoA, was produced as described previously [27] and used at 200 ng/ml. Raytide™EL, a general tyrosine kinase peptide substrate, and TAT-Pep5, a cell permeable p75^NTR ^signalling inhibitor were used at 1.0 μM, each purchased from Calbiochem (Darmstadt, Germany). [γ-^32^P]ATP was obtained from Perkin-Elmer (Madrid, Spain).

### Transfection vectors

The EGFP expressing vector (pEGFP-N1) was obtained from Clontech Laboratories, Inc. (Palo Alto, CA), while the pCDNA 3.1 Zeo encoding a HA-tagged form of wild type *(wt*) PTP1B was kindly provided by Carlos Arregui (Buenos Aires, Argentina) [58]. The pRK5-Myc vector encoding a Myc-tagged dominant negative form of RhoA, RhoA N19 (Addgene plasmid 15901) [26], was obtained from Addgene (Cambridge, MA).

### Cell cultures

Primary hippocampal neuron cultures were prepared as described previously [59], with some minor modifications. Briefly, the hippocampus was removed from E17 CD1 mouse foetuses and dissociated into single cells following trypsin (Worthington, Lakewood, USA) and DNase I digestion (Roche Applied Science). Neurons were plated on glass coverslips or in plastic dishes coated with poly-L-lysine (Sigma-Aldrich, Madrid, Spain), and cultured in Neurobasal A supplemented with 2 mM GlutaMAX I, 100 units/ml penicillin and 100 μg/ml streptomycin (Gibco BRL, Crewe, UK). After 7 days in vitro (DIV) the neurons were exposed to Aβ, NGF, and/or the pharmacological agents indicated. TrkA-deficient PC12 cells, PC12nnr5 [60], were grown in Dulbecco's modified Eagle's medium (DMEM) supplemented with 5% heat-inactivated foetal bovine serum (FBS), 10% heat-inactivated horse serum (Sigma-Aldrich), 2 mM GlutaMAX I, 100 units/ml penicillin and 100 μg/ml streptomycin (Gibco BRL). All cultures were kept at 37°C in a humidified atmosphere containing 5% CO_2_.

### Neuron transfection

Cultured neurons were transfected at 7 DIV with different vectors using the Effectene Transfection Reagent (Qiagen GmbH, Hilden, Germany) according to a modified version of the manufacturer's instructions. Briefly, 0.6 μg of DNA was added to 120 μl of the EC buffer and 3.5 μl of the Enhancer for each 35 mm cell culture dish of hippocampal neurons. The solution was incubated for 5 min at room temperature before 10 μl of Effectene was added, and after a further 15 min incubation at room temperature, the final solution was added to hippocampal neurons. The medium was then changed after 3 h.

### Immunocytochemistry, image acquisition and the morphometric analysis of labelled hippocampal neurons

At 16 h after transfection, the neurons were fixed for 30 min in 4% paraformaldehyde (PFA) prepared in PBS, they were then permeabilized for 15 min at room temperature with 0.5% Triton X-100 in PBS and blocked with 10% FBS in PBS containing 0.1% Triton X-100. The cells were incubated with the primary and secondary antibodies and the images of 10-20 neurons per coverslip were acquired digitally using a 63× oil immersion objective (Zeiss, Oberkochen, Germany). To analyze the dendrites, a region of interest (ROI) with a radius of 50 μm was projected onto EGFP-labelled neurons, its centre roughly coinciding with the centre of the soma. The dendrite length was expressed as the fraction of the dendritic tree that exceeds the limit of the ROI (fraction dendrites >50 μm). In co-transfection experiments, only double-labelled cells were analysed, which represented more than 90% of the single-labelled cells.

### Cell Survival

After treatment, the neurons were fixed for 30 min in 4% paraformaldehyde (PFA) in PBS and their nucleus was stained with the fluorescent dye, 4',6-diamidino-2-phenylindole (DAPI: Sigma-Aldrich). Non-viable neurons were recognized by nuclear condensation and/or the fragmentation of their chromatin. The number of viable neurons was counted in triplicate from ca. 50 fields in two independent experiments. In co-transfection experiments, only the nuclei of double-labelled cells were analysed.

### RhoA activation

To assay RhoA activation we followed a procedure described elsewhere [25]. Briefly, stimulated PC12nnr5 cells were first lysed in buffer: 50 mM Tris [pH 7.5], 500 mM NaCl, 10 mM MgCl_2_, 1.0% Triton X-100, 0.5% sodium deoxycholate, 0.1% SDS and a protease inhibitor cocktail (Roche Applied Science, Mannheim, Germany). The cleared lysates were incubated for 1 h at 4°C with Rhotekin-conjugated agarose beads (Cytoskeleton), and the beads were then collected by centrifugation and washed with the lysis buffer. Activated RhoA was detached from the beads by boiling for 5 minutes in Laemmli reducing buffer, after which it was immediately resolved by 12% SDS-PAGE and transferred to a nitrocellulose membrane. After blocking, the membranes were probed overnight at 4°C with a primary antibody directed against RhoA, which was detected with an HRP-conjugated secondary antibody that was visualised by enhanced chemiluminescence (GE-Healthcare, Piscataway, NJ). The intensity of the bands was evaluated by densitometry using ImageQuant software (GE-Healthcare).

### Protein phosphatase 1B assay

The PTP1B assay was performed essentially as described previously [61], with minor modifications. Briefly, 7DIV cultured hippocampal neurons (1 × 10^6 ^cells) were collected and homogenized in RIPA buffer (50 mM Tris [pH7.5], 150 mM NaCl, 2 mM EDTA, 1.0% Triton X-100 and an anti-protease cocktail). Equal amounts of protein were incubated for 2 h at 4°C with a mouse anti-PTP1B mAb, and then 20 μl of protein G sepharose was added and incubated for additional 2 h with agitation. Immunoprecipitated complexes were washed twice in RIPA buffer, once with the assay buffer (25 mM imidazole [pH7.2], 0.1 mg/ml BSA, 10 mM DTT) and they were then resuspended in 25 μl of assay buffer. The phosphatase substrate Raytide was labelled at its unique tyrosine residue with [γ-^32^P]ATP as described previously [62]. Assay mixtures (30 μl) containing the immunoprecipitated pellet and [^32^P]-labelled raytide (1 × 10^5 ^cpm) were incubated for 2 h at 30°C and the reaction was terminated by adding 750 μl of a charcoal mixture (0.9 M HCl, 90 mM sodium pyrophosphate, 2 mM NaH_2_PO_4_, 4% vol/vol Norit A). After centrifugation, the radioactivity in 400 μl of the supernatant was measured by scintillation counting. Blanks were determined by measuring the free [^32^P]phosphate in reactions where the immunoprecipitates were either boiled or omitted, and these values were subtracted from the reaction values.

### Quantitative real time polymerase chain reaction (PCR)

After treatment, the total RNA was extracted from cultures at 7 DIV using the Illustra RNAspin Mini kit (GE-Healthcare) and first strand cDNA was prepared from the RNA using the First Strand Synthesis kit (Fermentas GmbH, St Leon-Rot, Germany). Quantitative PCR was performed using the ABI Prism 7000 Sequence Detector (Applied Biosystems, Weiterstadt, Germany) and TaqMan probes. Primers for *Hes1 *and the housekeeping gene *GADPH *(as a control) were selected as the Assay-on-Demand gene expression products (Applied Biosystems). All TaqMan probes were labelled with 6-carboxy fluorescein (FAM) and real time PCR was performed using the TaqMan Universal PCR Master Mix according to the manufacturers' instructions. *Hes1 *expression was normalized to the *GAPDH *expression.

### Statistical analysis

Data are presented as the mean ± SEM and an unpaired Student's *t*-test was applied to determine the levels of significance, denoted as *p < 0.05, **p < 0.01, ***p < 0.001. All experiments were repeated at least twice.

## Competing interests

The authors declare that they have no competing interests.

## Authors' contributions

PJC performed the transfections, cultured the neurons, carried out the RT-PCR analysis and performed the immunocytochemistry for microscopy, as well as participating in the design and coordination of the work, and in the interpretation of data. RGM established the cultures of neurons and cell lines, performed the western blotting and immunocytochemistry, as well as participating in the analysis and interpretation of the data. ART designed the study, assayed the phosphatase activity, performed part of the microscopy analysis, analysed and interpreted the data, and drafted the manuscript.

All authors have read and approved the final manuscript.
